# Microstructure Sensitivity on Environmental Embrittlement of a High Nb Containing TiAl Alloy under Different Atmospheres

**DOI:** 10.3390/ma15238508

**Published:** 2022-11-29

**Authors:** Fan Zhang, Zeen Wu, Tiebang Zhang, Rui Hu, Xiaoye Wang

**Affiliations:** 1 Shannxi Province Key Laboratory of Advanced Manufacturing and Evaluation of Robot Key Components, Baoji University of Arts and Sciences, Baoji 721016, China; 2 State Key Laboratory of Solidification Processing, Northwestern Polytechnical University, Xi’an 710072, China

**Keywords:** high Nb containing TiAl alloy, mechanical properties, environmental embrittlement, microstructure sensitivity, synthesized system

## Abstract

Mechanical properties in different atmospheres, including oxygen, vacuum, air and H_2_, of high Nb containing TiAl alloys with the compositions of Ti–45Al–8.5Nb–(0.2W, 0.2B, 0.02Y) have been investigated in this work. Three different microstructure types, nearly lamellar, gamma phase increased nearly lamellar and fully lamellar are selected for revealing the microstructure sensitivity of environmental embrittlement. The results show that the three types of microstructures are all affected by the hydrogen–induced environmental embrittlement. Although the fracture mode of the experimental alloy is cleavage fracture in all atmospheres, the proportions of transgranular and intergranular fractures are different, especially comparing the fracture surfaces in oxygen and hydrogen. Performance comparison results show that the nearly lamellar microstructure is the most susceptible to the hydrogen–induced environmental embrittlement, while the gamma phase increased microstructure is the most stable one; the fully lamellar microstructure results in moderate susceptibility to the atmospheres. Combined with the hydrogen absorption kinetic analysis, it indicates that γ phase at the interface of lamellar colony significantly inhibits the hydrogen–induced environmental embrittlement, while the effect of β phase is just the opposite. In addition, the correlation between microstructure and hydrogen–induced environmental embrittlement is revealed and the corresponding mechanism is also discussed in this work.

## 1. Introduction

TiAl alloys have attracted more and more attention due to their low density, high specific strength and stiffness, good oxidation resistance and high temperature creep resistance [1,2,3,4]. Hence, TiAl alloys have wide application prospects in high–temperature resistant components such as low–pressure turbine blades of aero–engines, high–pressure compressor blades, turbine augmenters and the new generation nuclear reactors [5,6]. The elastic modulus and creep behavior of TiAl alloys are better than titanium alloys and equivalent to Ni–based superalloys [2]. Additionally, the density of a TiAl alloy is less than half of a superalloy. Meanwhile, the service temperature of a TiAl alloy can reach 650~800 °C, which can fill the gap of titanium alloys and superalloys [7]. Consequently, the TiAl alloy is considered as having the most application potential for the new generation of aerospace lightweight high–temperature structural materials [2,7]. However, the large–scale application of the TiAl alloy is still limited by many aspects. The brittleness of a TiAl alloy has seriously hindered its application in aerospace and other important fields [8,9]. 

The brittleness of a TiAl alloy can be divided into intrinsic brittleness and environmental embrittlement. In view of the intrinsic brittleness problem, researchers have done a lot of effective work in composition design, microstructure control and the structure–activity relationship, which have significantly improved mechanical properties of TiAl alloys [10,11]. In the research of alloying, the addition of niobium (chemical symbol Nb, atomic number 41) is widely covered. The studies show that the service temperature of the high Nb containing TiAl alloy can be increased by 60–100 °C and the high–temperature yield strength can be doubled, compared with the ordinary TiAl alloy [12]. Meanwhile, the high–temperature oxidation resistance has reached the level of the nickel-based superalloy for turbine disk [13,14]. Therefore, the high Nb containing TiAl with excellent high–temperature performance has become one of the most important development directions of the TiAl alloy [15]. In addition to intrinsic brittleness, the main reason for low plasticity is environmental embrittlement. Hydrogen embrittlement is the most important form of reducing the mechanical properties of alloys, and has been one of the research hot spots in the field of TiAl alloys for many years [16,17]. 

Research shows that the hydrogen–induced environmental embrittlement of a TiAl alloy can be affected by many factors. Hotta et al. tested five TiAl alloys with different compositions and found that all alloys showed reduced fracture strength and fracture strain in air, water vapor and H_2_ + Ar compared to vacuum, but exhibited different sensitivities [18]. The different compositions reflect the difference in phase content in the alloy. Further research shows that the hydrogen absorption ratio of α_2_ phase is larger than γ phase [19]. Therefore, TiAl alloys with the same composition can change the ratio of phase content through reasonable processes to achieve the best effect of hydrogen embrittlement resistance. Moreover, different microstructure types exhibit variability in environmental brittleness resistance [20]. Research shows that the hydrogen embrittlement resistance of single γ structure is best, while duplex is the worst. For the fully lamellar microstructure, the effect of hydrogen embrittlement resistance increases with the decrease of lamellar spacing [21]. In addition to the influence of microstructure, alloying elements also play a positive role in resisting environmental embrittlement [22,23]. The precipitates produced by alloying elements can improve the hydrogen embrittlement resistance of steel material [24]. However, there is no such report in the field of TiAl alloys.

Although there have been many studies on hydrogen embrittlement, there are still problems in composition selection and microstructure control of TiAl alloys. Meanwhile, it is worth noting that the study of hydrogen embrittlement is still limited to the conventional TiAl alloys, while the environmental embrittlement behavior and the corresponding mechanism of high Nb containing TiAl alloys with promising engineering applications have not been well illustrated in the literature. Furthermore, when discussing the environmental sensitivity of microstructure types, scholars often choose different alloy compositions. However, composition also affects the environmental sensitivity of the alloy. Therefore, it is more reasonable to choose one alloy composition with different microstructure types to study the environmental sensitivity. In this study, the hydrogen–-induced environmental embrittlement of high Nb containing TiAl alloy is investigated by testing its properties under different atmospheres. The sensitivity of microstructure to hydrogen embrittlement and the corresponding mechanism are discussed by selecting different microstructure types of the experimental alloy. It is expected that this work can provide theoretical basis and technical guidance for the practical application of lightweight and high strength TiAl alloys.

## 2. Experimental Procedures

**Figure 1:**
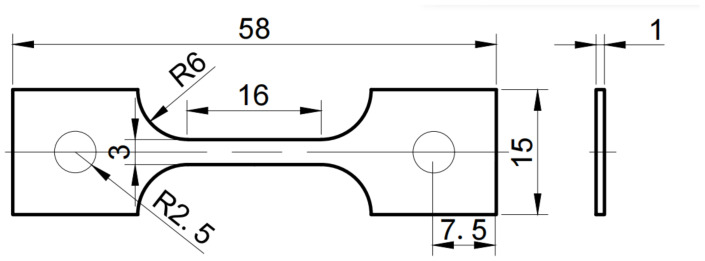
Geometry of the tensile specimen.

The experimental alloy with a nominal composition of Ti–45Al–8.5Nb–(0.2W, 0.2B, 0.02Y) was selected in this work. The alloy composition was chosen based on the desired properties of Ti–45Al–(5–10)Nb. Meanwhile, tungsten, boron and yttrium are the alloying elements that significantly improve the comprehensive performance of TiAl alloy. The ingot (500 mm× 200 mm × 200 mm) was prepared by twice plasma arc melting (PAM) to homogenize the composition, with the commercial high–purity starting materials of Al (99.97 wt%), Ti (99.97 wt%), Ti–Nb binary alloy (52.47 wt% Nb), W (99.9 wt%), TiB_2_ (99.5 wt%) and Y (99.9 wt%). The heat treatment experiment was carried out in a high–temperature heat treatment furnace. In order to obtain different microstructure types, 1280 °C in the α phase region and 1240 °C in the α + γ phase region are chosen for heat treatment based on the Ti–Al–Nb phase diagram [25]. Two samples taken from the ingot with dimensions of 60 mm × 20 mm × 20 mm were annealed in vacuum tubes at 1240 °C for 6 h and 1280 °C for 9 h, respectively. Together with the as–cast alloy, three types of microstructures were subjected to atmospheric tensile testing. The skin of the heat–treated sample was removed and then processed according to the tensile sample size. Three tensile specimens were prepared by electrical discharge machining (EDM) for each atmosphere, with the size of 58 mm × 15 mm × 1 mm in Figure 1. The size of tensile specimen was mainly determined by the atmosphere chamber size of mechanical testing machine. The measurement error in tensile testing was defined using the relative standard deviation. All the samples were prepared by standard metallographic polishing procedures.

Due to TiAl alloys being brittle materials, the fracture strength and plastic elongation to failure (ε_f_) of the ultrasonic cleaned samples were tested in oxygen, vacuum, air and hydrogen atmospheres. The tensile tests in hydrogen and oxygen atmospheres were performed on an MTS mechanical testing machine equipped with an atmospheric chamber. Before the experiment, the atmospheric chamber was vacuumized and then filled with the corresponding gas. After standing for ten minutes, the tensile tests were performed at a strain rate of 0.2 mm min^−1^. The microstructure, phase composition and fracture morphology of the alloy were observed and analyzed by a scanning electron microscope (SEM) equipped with energy dispersive spectroscopy (EDS). At least 5 areas were randomly selected for each observation. Samples for SEM observation were etched using a modified Kroll’s etchant consisting of 40 mL H_2_O, 5 mL HNO_3_ and 5 mL HF. After ultrasonic, acetone and alcohol cleaning, the microstructure of corrosion samples was observed. The phase constitutions of the samples were determined by X–ray diffraction (XRD) with Cu Kα radiation (λ = 1.54056 Å). The phase content of the alloy was determined by Rietveld analysis of XRD patterns [26]. The hydrogen absorption experiment was carried out in a PCTPro E&E high-pressure gas adsorption and desorption analyzer at 350 °C and 7 MPa hydrogen pressure for 1 h.

## 3. Results

### 3.1. Phase Composition and Microstructure Evolution 

**Figure 2:**
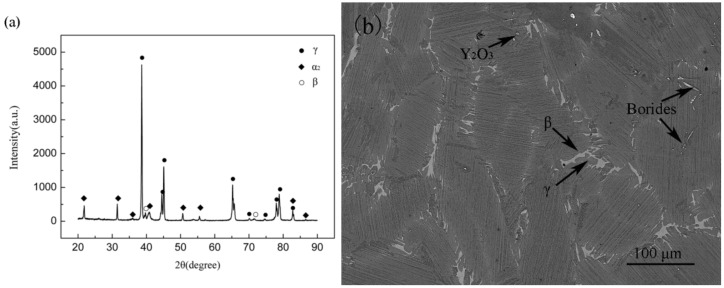
(**a**) XRD pattern and (**b**) SEM image of the Ti–45Al–8.5Nb–(0.2W, 0.2B, 0.02Y) alloy.

Figure 2a is the XRD pattern of Ti–45Al–8.5Nb–(0.2W, 0.2B, 0.02Y) alloy. The main phases in the alloy are α_2_ and γ. The lower intensity of the diffraction peaks of the β phase indicates less content relative to the main phase. No Nb compound phase is found in the XRD pattern, indicating that the Nb element is dissolved in the alloy matrix. The report also confirms that Nb elements occupy the positions of Ti atoms in the matrix [27]. The existence of β phase is related to the non–equilibrium solidification path of the experimental alloy, as shown in the Ti–Al–Nb phase diagram [25]. In addition, since Nb and W are β stabilizing elements, they can lower the β to α transition temperature and expand the β phase region [13]. 

Figure 2b shows the BSE–SEM microstructure of the experimental alloy. It can be seen that the microstructure is composed of relatively uniform and fine lamellar colonies. According to the EDS analysis results in Table 1, the white contrasting phase located between and inside the lamellar colonies is the residual β phase. The dark contrast next to the β phase between the lamellar colonies is the γ phase. The reason for the formation of this microstructure is due to the β–solidification of the experimental alloy. During the solidification process of the alloy, α phase with different orientations can be formed through the transformation from β to α. Therefore, the microstructure of the alloy shows that the residual β phase wraps around the α phase with different orientations [28]. In the subsequent solid–state transformation process, the γ phase transforms from the α phase [29], and a fine γ + (α_2_ + γ) lamellar microstructure is formed. According to energy spectrum analysis, borides and Y_2_O_3_ precipitates are also present in the alloy, which are randomly distributed as particles or strips. In addition, yttrium oxides are also detected by EDS.

**Figure 3:**
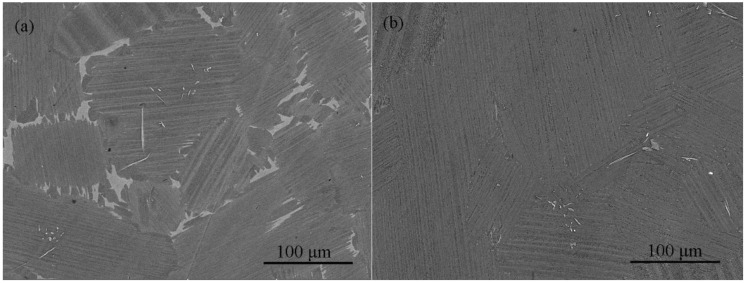
SEM images of Ti–45Al–8.5Nb–(0.2W, 0.2B, 0.02Y) alloy: (**a**) annealed at 1240 °C for 6 h, (**b**) annealed at 1280 °C for 9 h.

In this work, two different heat treatments are performed on the as–cast alloy, as shown in Figure 3. Compared with the as–cast alloy, the alloy annealed at 1240 °C presents an increased γ phase and decreased β phase on the colony boundaries. After heat treatment at 1280 °C, the microstructure type changes to full lamellar. The three alloy microstructures are divided into two microstructure types: near lamellar and full lamellar microstructure. The microstructure type, colony size and phase content of the three alloys have been explained in the authors’ previous work [17]. It is known from the previous work that the similar colony sizes of the three microstructures can ensure that they are in a comparable range. The three microstructure types are called nearly lamellar, γ phase increased nearly lamellar and fully lamellar in subsequent expressions. 

### 3.2. Mechanical Properties and Fracture Feature of the As–Cast Alloy 

**Figure 4:**
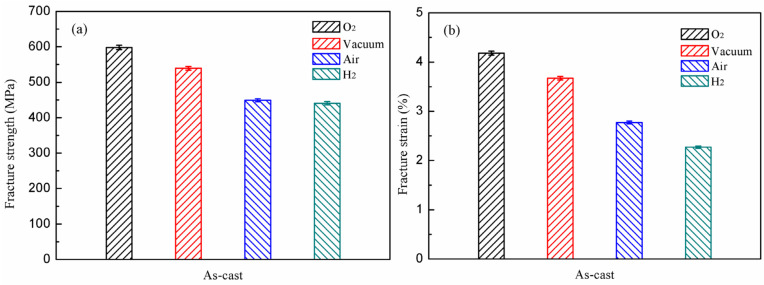
The mechanical properties of as–cast Ti–45Al–8.5Nb–(0.2W, 0.2B, 0.02Y) alloy under different atmospheres: fracture strength (**a**); ε_f_ (**b**).

Figure 4 displays the tensile properties of as–cast experimental alloy in oxygen, vacuum, air and hydrogen atmospheres, respectively. Charging oxygen into the evacuated experimental chamber will hinder the environmental embrittlement caused by the contact of TiAl’s surface with water vapor and hydrogen. Therefore, it is generally believed that the mechanical properties of the TiAl alloy under oxygen environment are its intrinsic properties. As seen in Figure 4, in the oxygen atmosphere, the fracture strength and fracture strain of the experimental alloy are the highest among the four environmental media, at 598.4 MPa and 4.18%, respectively. In the vacuum, a small amount of air will be present in the laboratory due to the limitations of the vacuum degree. Additionally, the presence of water vapor in the air can affect the experimental alloys, resulting in environmental embrittlement [17]. Consequently, its fracture strength and fracture strain are decreasing at 539.6 MPa and 3.67%, respectively. Compared with the performance of the alloy in oxygen, the fracture strength and fracture strain under vacuum conditions are reduced by 9.83% and 12.2%, respectively. 

Compared to vacuum, the increased water vapor content in the air, and possibly a little hydrogen, leads to a significant reduction in alloy properties of 449.1 MPa and 2.77%, respectively. Additionally, the further decrease in percentage of fracture strength and fracture strain properties in the air is 7.5% and 24.5%, respectively. It can be seen that the alloy is more obviously affected by the environmental embrittlement factors in the air. In the hydrogen atmosphere, the alloy exhibits the lowest fracture strength and fracture strain, at 441.0 MPa and 2.27%, respectively. Compared with the air, the performance degradation ratio of the alloy in the hydrogen atmosphere is as follows: the fracture strength is 11.6%, and the fracture strain is 18.1%. From the authors’ previous work [17], comparing with the reduction ratio of the as–cast alloy in the water vapor and air, it indicates that hydrogen has a greater effect on the experimental alloy. The degradation of the alloy’s properties tends to accelerate with the changing atmospheres. It means that the alloy is very sensitive to the content of hydrogen and water vapor in the environment, and the properties will change significantly with the increase of their content.

**Figure 5:**
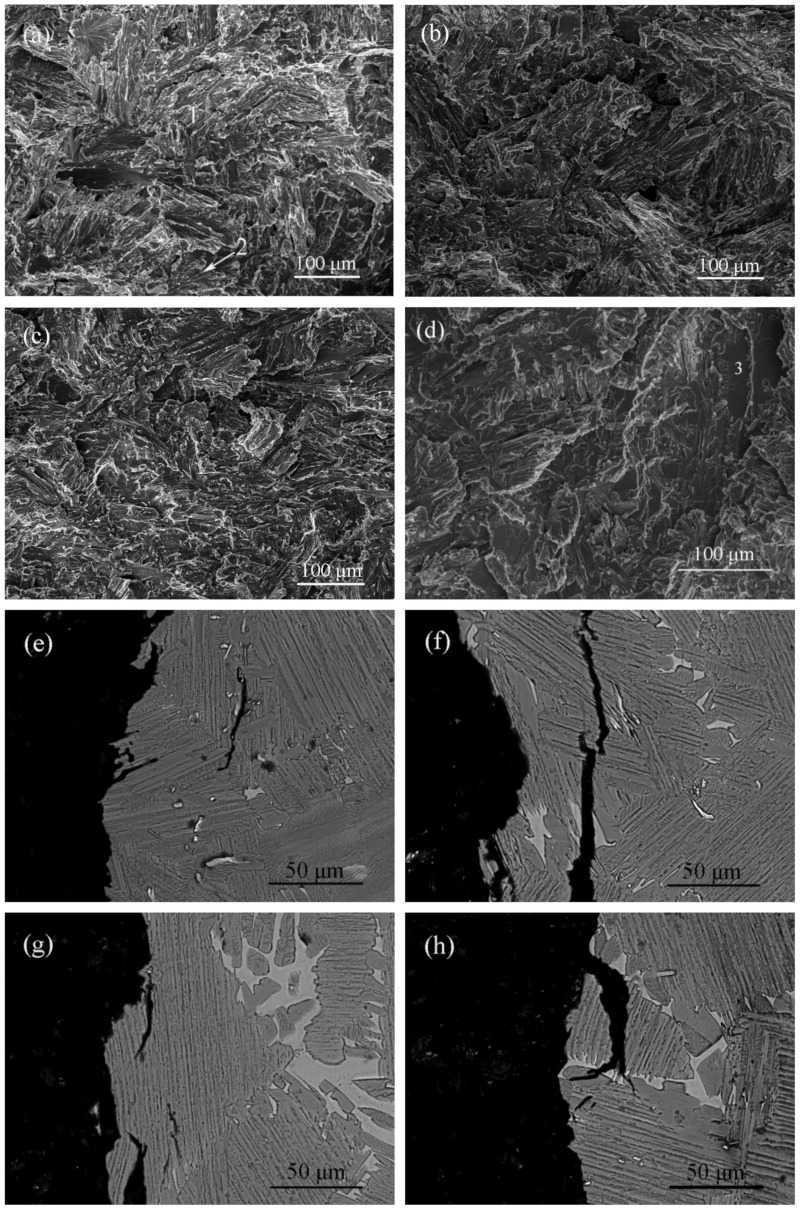
SEM images of fracture surfaces for as–cast alloy after tensile test in: (**a**,**e**) oxygen; (**b**,**f**) vacuum; (**c**,**g**) air; (**d**,**h**) hydrogen. “1” translamellar fracture surface, “2” river patterns and “3” interlamellar fracture surface.

The fracture morphologies of the as–cast alloy in the four atmospheres are shown in Figure 5a–d. It can be seen in Figure 5a that the fracture of the alloy in the oxygen environment exhibits river–like patterns revealing the predominance of a transgranular cleavage fracture, accompanied by a small amount of smooth facets revealing intergranular fractures. In the other three atmospheric environments, their fracture modes are also cleavage fractures, as shown in Figure 5b–d. However, in oxygen and vacuum atmospheres, the results show a high percentage of transgranular fractures, indicating that the alloy fracture is the intrinsic or close to the intrinsic fracture. In the air and hydrogen, as shown in Figure 5c,d, although the fracture process of the alloy is still dominated by transgranular fractures, the proportion of intergranular fractures increases significantly. Especially in the hydrogen, the fracture exhibits a mix of inter–and trans–lamellar fractures. That is, the proportion of intergranular fractures in the fracture process under the hydrogen is higher than that of oxygen, which indicates that the effect of hydrogen embrittlement mainly affects intergranular fractures. Hydrogen reduces the bonding force between the lamellar colonies, leading to the fracture failure process in the experimental alloy. All of the main testing details and results are shown in Table 2.

In order to investigate the process of crack initiation and propagation during alloy fracture, the profile images of the fracture specimens in different atmospheres are displayed in Figure 5e–h. It can be seen that the crack initiation positions are mainly between boride or Y_2_O_3_/matrix in oxygen, as shown in Figure 5e. Since borides and Y_2_O_3_ are randomly distributed in the interior of the lamellar, the cracks will propagate from the interior of the lamellar to the adjacent one, resulting in a very high proportion of transgranular fracture. Figure 5f shows the microstructure of the fracture surface in the vacuum. In addition to the same crack initiation sites as the oxygen environment, it is also found that the cracks propagate along the colony boundaries, and are mainly concentrated between the β phase/matrix. The crack nucleation between the β phase/matrix is more pronounced in the air and hydrogen environment in Figure 5g,h. This makes it more likely that the cracks will propagate along the colony boundary, which in turn increases the proportion of intergranular fracture during the fracture process.

### 3.3. Mechanical Properties and Fracture Feature of the Alloy Annealed at 1240 °C

**Figure 6:**
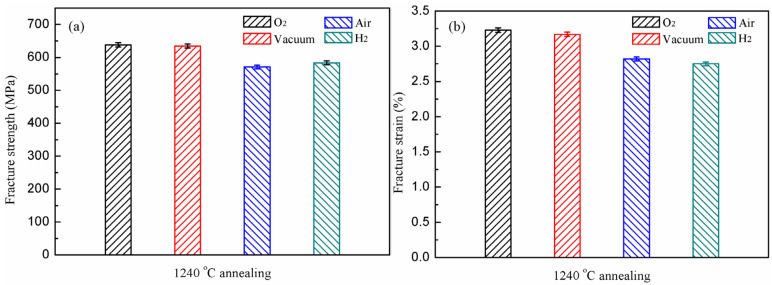
The mechanical properties of 1240 °C annealed Ti–45Al–8.5Nb–(0.2W, 0.2B, 0.02Y) alloy under different atmospheres: fracture strength (**a**); ε_f_ (**b**).

The tensile properties of the experimental alloy annealed at 1240 °C in different atmospheres are illustrated in Figure 6. It can be seen that the experimental alloy has the highest fracture strength and fracture strain in oxygen with 637.8 MPa and 3.23%, respectively. Compared to the performance of the alloy in the oxygen environment, the fracture strength and fracture strain of the alloy in vacuum are reduced by 0.49% and 1.9%, respectively. This is different from the performance characteristics of the as–cast alloy in the two atmospheres. After heat treatment, the properties of the experimental alloy in oxygen and vacuum environments are only slightly different, which indicates that the alloy is less affected by a small amount of water vapor environment after being annealed at 1240 °C.

The performance of the alloy decreases significantly in the air, and its fracture strength and fracture strain are 571.2 MPa and 2.82%, respectively. Compared to the vacuum environment, the property degradation percentages of the alloy in air are 10% in fracture strength and 11% in fracture strain. Comparing with the properties of the as–cast alloy, this indicates that the 1240 °C annealed alloy is more resistant to the water vapor environment than the as–cast one. The performance of the alloy in hydrogen reveals some subtle differences from that in air. The fracture strength and fracture strain of the alloy are 583.8 MPa and 2.75%, respectively. The fracture strength of the alloy in the hydrogen environment increases slightly, and the fracture strain decreases by 2.5%. It reveals that the 1240 °C annealed alloy is less affected by the hydrogen. In other words, the γ phase increased nearly lamellar microstructure has a significant resistance to the hydrogen embrittlement.

**Figure 7:**
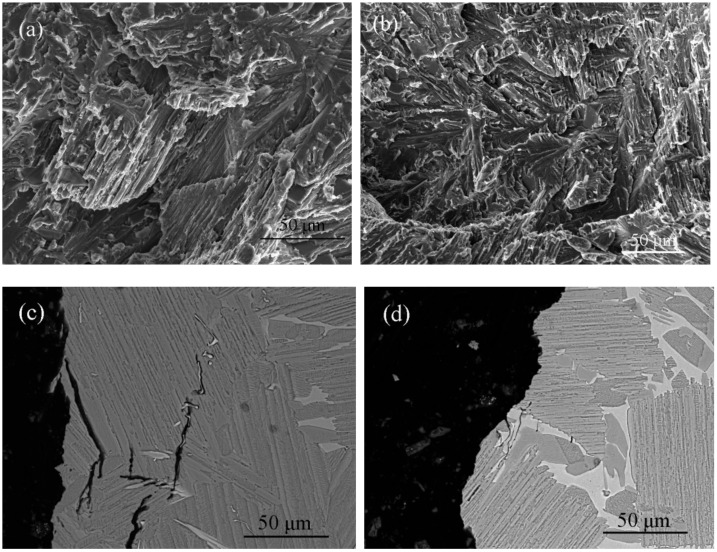
SEM images of fracture surfaces for 1240 °C annealed alloy after tensile test in: (**a**,**c**) oxygen; (**b**,**d**) hydrogen.

Since 1240 °C annealed alloy has the same microstructural characteristics as the as–cast alloy, its fracture morphology is similar. It can be seen from the above that the fracture modes have little difference in the four atmospheres. Therefore, only two types of fracture structures with greater variability in oxygen and hydrogen are selected for analysis. As shown in Figure 7a, the alloy exhibits its intrinsic brittle cleavage fracture with a river–like pattern in the oxygen, which is characterized by transgranular fractures as the main, and intergranular fractures as the secondary feature. Since the fracture characteristics are all cleavage fractures, there is no obvious difference in the fracture of the alloy between oxygen and hydrogen atmospheres. However, the percentage of intergranular fracture is slightly increased for hydrogen compared to oxygen. This indicates that the effect of hydrogen–induced environmental embrittlement on this microstructure is less. All of the main testing details and results are shown in Table 3.

Figure 7c,d presents the fracture profile images of the 1240 °C annealed specimens in oxygen and hydrogen. In the oxygen, as shown in Figure 7c, the main crack initiation sites are between borides or the Y_2_O_3_/matrix. Since borides and Y_2_O_3_ are mainly distributed inside the lamellar colonies, this leads to transgranular fractures, resulting the high proportion of transgranular fractures in the fracture process. Figure 7d is the image of the fracture surface in hydrogen. The crack initiation is more obvious between the β phase/matrix, which increases the probability of the alloy crack propagation along the interface of the lamellar and leads to the increasing proportion of intergranular fractures. However, the initiation of cracking is not found inside the γ phase or between the matrix/γ phase.

### 3.4. Mechanical Properties and Fracture Features of the Alloy Annealed at 1280 °C

**Figure 8:**
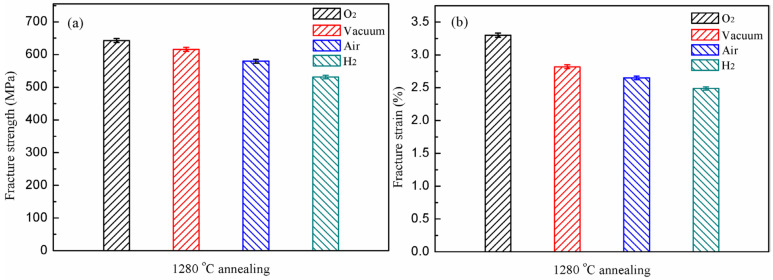
The mechanical properties of 1280 °C annealed Ti-45Al-8.5Nb-(0.2W, 0.2B, 0.02Y) alloy under different atmospheres: fracture strength (**a**); ε_f_ (**b**).

Figure 8 illustrates the tensile properties of the experimental alloy annealed at 1280 °C in the four atmospheres. The experimental alloy in the oxygen environment has the highest fracture strength and fracture strain at 642.8 MPa and 3.3%, respectively. Compared with the other two microstructure types, the fully lamellar alloy has the highest fracture strength in oxygen. In the vacuum, the fracture strength and fracture strain of the alloy are 616.1 MPa and 2.82%, respectively. It exhibits different performance characteristics to the 1240 °C annealed alloy. In the vacuum, its properties, especially the fracture strain, decreased significantly, indicating that the fully lamellar alloy is very sensitive to the environment. In the air, the performance of the alloy decreases significantly, and its fracture strength and fracture strain are 579.5 MPa and 2.65%, respectively. It indicates that with the increase of water vapor and hydrogen content in the air, the fully lamellar microstructure is further affected by the environment and leads to the decline of the properties. The fracture strength and fracture strain of the alloy in hydrogen are 531.1 Mpa and 2.49%, respectively. Its performance is further degraded. It suggests that the fully lamellar alloy is greatly affected by the hydrogen environment. In the four atmospheres, the decrease of the fracture strength shows an accelerated trend, but the fracture strain is not as affected. It implies that the fully lamellar alloy is highly sensitive to the content of water vapor and hydrogen in the environment.

**Figure 9:**
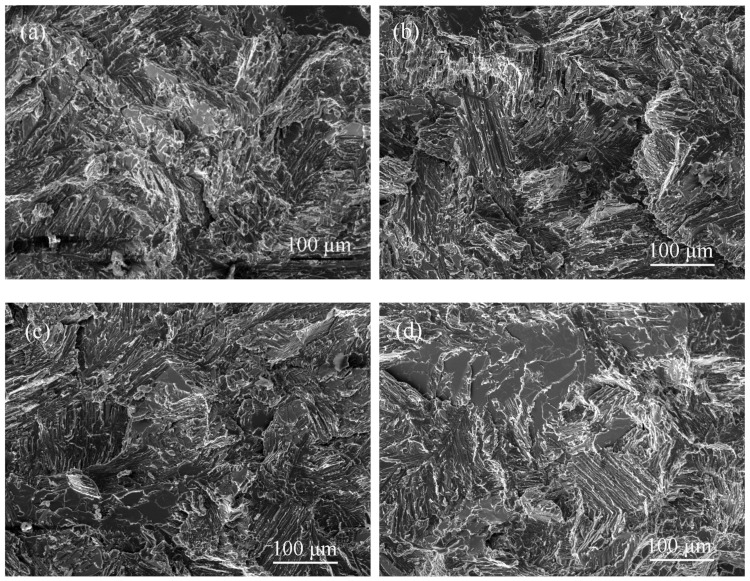

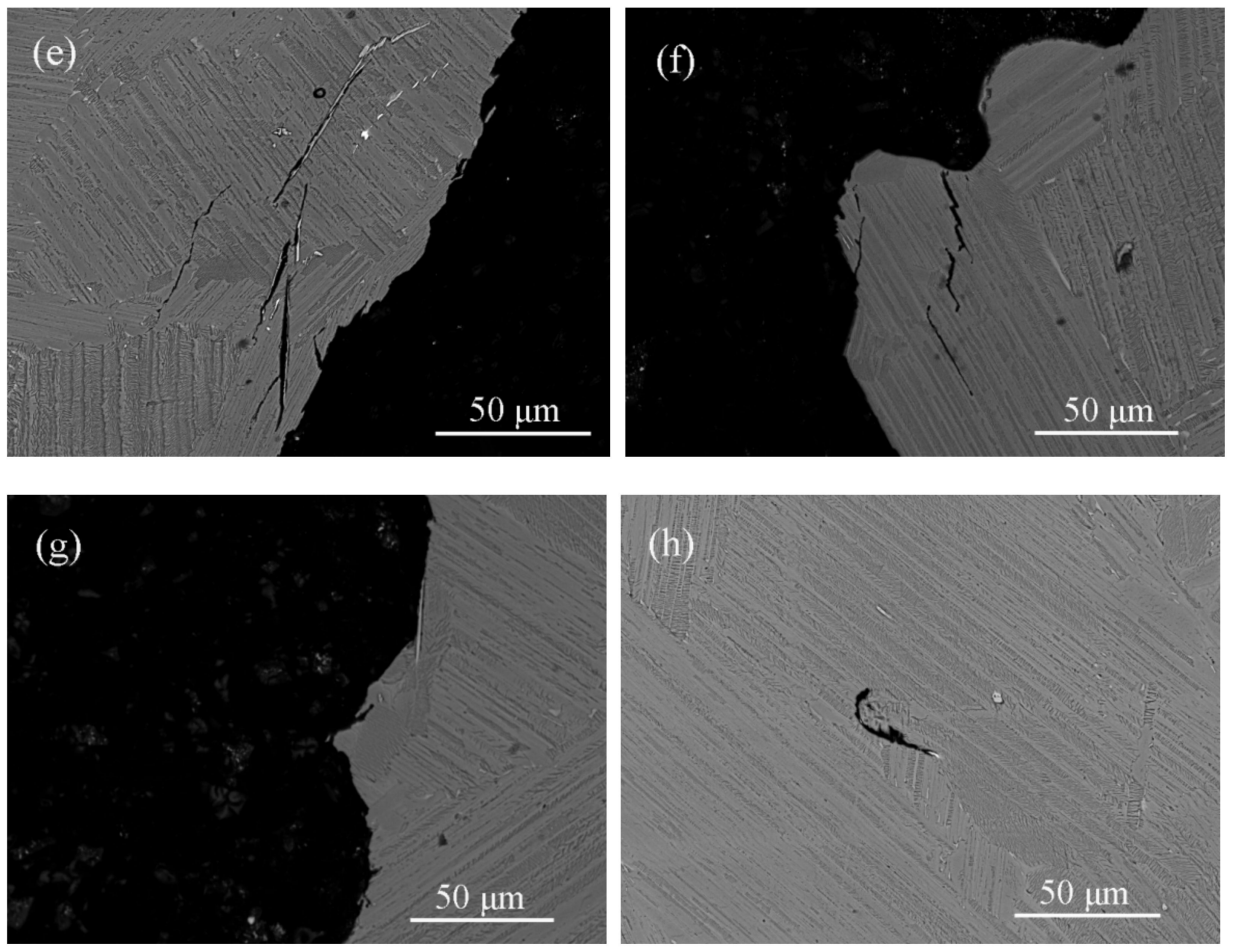
SEM images of fracture surfaces for 1280 °C annealed alloy after tensile test in: (**a**,**e**) oxygen; (**b**,**f**) vacuum; (**c**,**g**) air; (**d**,**h**) hydrogen.

Figure 9 shows the fracture morphologies of the fully lamellar alloy in the four atmospheres. As shown in Figure 9a, the fracture mode of the alloy in the oxygen environment is a cleavage fracture dominated by transgranular fractures. It can be seen from Figure 9b–d that the fracture characteristics of the experimental alloy in the other three atmospheres are also cleavage fractures. Although the fracture mode is still dominated by transgranular fractures, the proportion of intergranular fractures is gradually increasing. Especially in the hydrogen, the fracture exhibits a mix of inter-and trans–lamellar fractures. It indicates that the effect of hydrogen–induced environmental embrittlement on the fully lamellar alloy is most significant. All of the main testing details and results are shown in Table 4.

The profile images of the alloy fracture surfaces in different atmospheres after 1280 °C annealing are shown in Figure 9e–h. Since the microstructure type of the alloy is fully lamellar, the crack initiation positions are different from that of the former two. In the four atmospheres, the crack initiation position has no significant change. The main initiation positions of the alloy cracks are inside the lamellar colonies and between the boride or Y_2_O_3_/matrix. As a result, the crack propagates toward the adjacent lamellar colony, resulting in a transgranular fracture. When borides and Y_2_O_3_ precipitates are present at the interface, the crack propagates along the colony boundary and then causes an intergranular fracture during the fracture process. However, the increase of the intergranular fracture ratio cannot be seen from the profile of the fracture surface.

## 4. Discussion

Studies have shown that most ordered intermetallic compounds have low resistance to environmental embrittlement, but TiAl is better in comparison, especially high Nb containing TiAl alloys [30,31]. In addition to the nature of the TiAl alloy, the alloying elements also play a key role. The alloying element Nb is reported to increase the dissociation energy Gc of Ti_3_Al alloy while weakening the Ti–Ti covalent bond, thus improving the intrinsic brittleness of Ti_3_Al [32]. This suggests that the presence of alloying elements inhibits hydrogen embrittlement in two ways. On the one hand, the presence of alloying elements may hinder the entry of hydrogen atoms by competing for adsorption, occupying more vacancies and grain boundaries. On the other hand, it can also influence its essential properties.

By comparing the performance of the three selected microstructure types in different atmospheres, it can be found that all three types suffer from significant hydrogen–induced environmental embrittlement, indicating that any microstructure type of the alloy is subject to environmental influences. The mechanism of hydrogen–induced environmental embrittlement on experimental alloys mainly includes two aspects. First, hydrogen reduces the stress required for the onset of plasticity by reducing the shear modulus, dislocation line energy and laminar dislocation energy [33]. The second is the catalytic aggregation of hydrogen. In the process of plastic deformation, the hydrogen ions generated by the catalytic Al element are transported to the crack tip by moving dislocations and diffusing along the pipes of the dislocation nucleus [34,35,36]. Meanwhile, dislocations, grain boundaries, phase interfaces and crack surfaces, which connect to the free surface, can provide fast diffusion paths for hydrogen absorbed from the material surface [37,38]. Therefore, the local accumulation of hydrogen is considered to cause the reduction of elongation, resulting in hydrogen–induced environmental embrittlement.

However, there are significant differences in the sensitivity of the three microstructure types to the hydrogen atmosphere. Since the alloy exhibits intrinsic brittleness in the oxygen environment and its worst performance in the hydrogen environment, Table 5 shows the proportion of mechanical property degradation of the three experimental alloys in the hydrogen compared with the oxygen. It shows that the as–cast alloy is the most susceptible to hydrogen-induced environmental embrittlement. The performance of the γ phase increased nearly lamellar alloy is the most stable one in both atmospheres, indicating that the alloy has significant hydrogen embrittlement resistance. Additionally, the fully lamellar alloy results in moderate susceptibility to the hydrogen-induced environmental embrittlement.

Since the three resulting microstructures come from different thermal treatments, it can be inferred that the different environmental sensitivities of the three alloys are determined by the microstructure types. For the as–cast alloys and the 1240 °C annealed alloy, the main difference between them is the increase of the γ phase and the decrease of the β phase at the interface of the lamellar colonies. It has been reported that the phase morphology has a greater impact on environmental embrittlement than the content [39]. The performance comparison indicates that the existence of γ phase at the interface of the lamellar has a significant inhibitory effect on the hydrogen–induced environmental embrittlement. Meanwhile, the first principles calculations show that the γ phase easily adsorbs hydrogen, making it difficult to diffuse, thereby inhibiting the propagation of hydrogen and reducing the impact of hydrogen–induced environmental embrittlement [40]. Therefore, it can be concluded that the blockish shape γ phase at the interface of the lamellar colonies can significantly inhibit the hydrogen–induced environmental embrittlement process of the experimental alloy.

**Figure 10:**
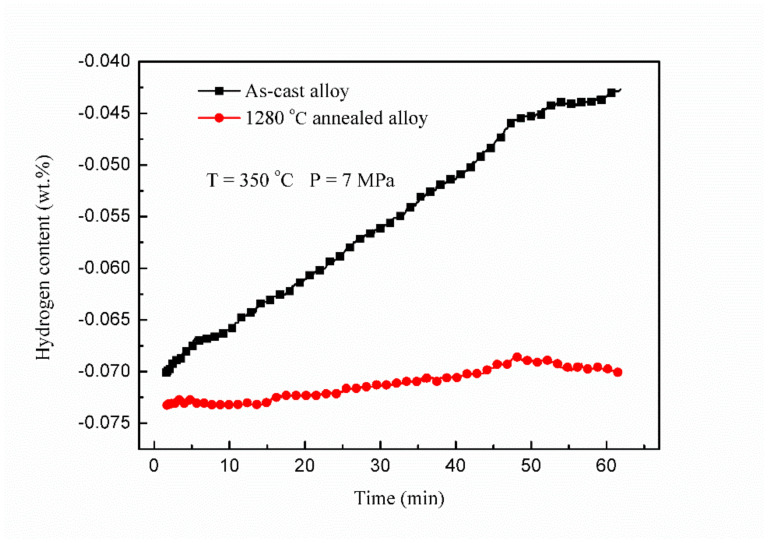
Hydrogen absorption curves under an initial hydrogen pressure of 7 MPa of as–cast alloy (black line) and the alloy annealed at 1280 °C (red line).

The performance of the fully lamellar alloy is more stable than the as–cast alloy, indicating that although the inhibitory effect of γ relative to hydrogen embrittlement disappears after heat treatment, the disappearance of β phase also slows down the brittle behavior process of the alloy under hydrogen. It illustrates that β phase is very sensitive to the hydrogen and promotes the hydrogen–induced environmental embrittlement process of the alloy. To further confirm this point, the hydrogen absorption kinetics of as–cast and fully lamellar alloys at 350 °C and 7 MPa hydrogen pressure are studied, as shown in Figure 10. It can be found that the hydrogen absorption capacity of as–cast alloy is much higher than that of fully lamellar one. Since the inhibitory effect of γ on hydrogen embrittlement has been demonstrated, it can be revealed that the β phase has a role in promoting hydrogen–induced environmental embrittlement. To sum up, for high Nb containing TiAl alloys with promising engineering applications, hydrogen–induced environmental embrittlement is very sensitive to γ and β phases existing at the interface of the lamellar colonies. It can be deduced that an appropriate amount of blockish shape γ phase and as little β phase as possible in high Nb containing TiAl alloys is one of the key factors for the alloy with maximum environmental embrittlement resistance.

## 5. Conclusions

In this work, the mechanical properties of the Ti–45Al–8.5Nb–(0.2W, 0.2B, 0.02Y) alloy in oxygen, vacuum, air and hydrogen atmospheres are investigated. Meanwhile, the microstructure sensitivity of hydrogen-induced environmental embrittlement in the alloys is revealed by the selected three microstructure types. The main conclusions are as follows. It is also necessary to note that these conclusions are only the preliminary results and that the more tests are needed to confirm the indications outlined form this experimental work.

(1) The mechanical properties of all the three microstructure types show a gradual declining trend in the four environmental atmospheres, indicating that each microstructure can be affected by environmental embrittlement. The γ phase increased nearly lamellar microstructure is the most resistant to hydrogen–induced environmental embrittlement. The as–cast alloy is the most susceptible to environmental embrittlement. In addition, the fully lamellar microstructure results in moderate susceptibility to hydrogen–induced environmental embrittlement.
(2) The fracture modes of the three microstructure types in the four atmospheres have not changed. However, although the transgranular fracture is dominant, the proportion of intergranular fractures increases in air and hydrogen. It indicates that hydrogen embrittlement mainly acts on the intergranular fracture, which reduces the bonding force between the lamellar colonies, resulting in the low ductility of the alloy.
(3) Hydrogen–induced environmental embrittlement is very sensitive to γ and β phases existing at the interface of the lamellar colonies for the experimental alloy. The γ phase can inhibit the hydrogen–induced environmental embrittlement, while β phase is just the opposite. It is disclosed in the present study that an appropriate amount of blockish shape γ phase and as little β phase as possible in high Nb containing TiAl alloy is one of the key factors for creating the alloy with maximum environmental embrittlement resistance.

## Figures and Tables

**Table 1:** EDS analysis of the phases in Figure 1 (at %).

Phases	Ti	Al	Nb	W	B	Y	O
β	51.85	33.98	13.41	0.75	_	_	_
Boride	24.53	7.12	9.24	0.54	58.58	_	_
Yttrium oxide	20.9	17.08	_	_	_	14.65	47.37

**Table 2:** The main testing details and results of as–cast alloy under different atmospheres.

Specimen	Heat Treatment	Tested Environment	Tensile Properties		Main Metallografic Findings	
			Fracture Strength (MPa)	εf (%)	Fracture Type	Fracture Mode
1, 2, 3	As-cast	O_2_	598.4	4.18	Cleavage fracture	Transgranular fracture (predominant) Intergranular fracture (secondary feature)
4, 5, 6	As-cast	Vacuum	539.6	3.67	Cleavage fracture	Transgranular fracture (predominant) Intergranular fracture (slightly increased)
7, 8, 9	As-cast	Air	499.1	2.77	Cleavage fracture	Transgranular fracture (predominant) Intergranular fracture (increase in proportion)
10, 11, 12	As-cast	H_2_	441.0	2.27	Cleavage fracture	Mix of inter–and trans–lamellar fracture

**Table 3:** The main testing details and results of 1240 °C annealed alloy under different atmospheres.

Specimen	Heat Treatment	Tested Environment	Tensile Properties		Main Metallografic Findings	
			Fracture Strength (MPa)	εf (%)	Fracture Type	Fracture Mode
1, 2, 3	Annealed at 1240 °C	O_2_	637.8	3.23	Cleavage fracture	Transgranular fracture (predominant) Intergranular fracture (secondary feature)
4, 5, 6	Annealed at 1240 °C	Vacuum	634.7	3.17	Cleavage fracture	Transgranular fracture (predominant) Intergranular fracture (secondary feature)
7, 8, 9	Annealed at 1240 °C	Air	571.2	2.82	Cleavage fracture	Transgranular fracture (predominant) Intergranular fracture (slightly increased)
10, 11, 12	Annealed at 1240 °C	H_2_	583.8	2.75	Cleavage fracture	Transgranular fracture (predominant) Intergranular fracture (slightly increased)

**Table 4:** The main testing details and results of 1280 °C annealed alloy under different atmospheres.

Specimen	Heat Treatment	Tested Environment	Tensile Properties		Main Metallografic Findings	
			Fracture Strength (MPa)	εf (%)	Fracture Type	Fracture Mode
1, 2, 3	Annealed at 1280 °C	O_2_	642.8	3.30	Cleavage fracture	Transgranular fracture (predominant) Intergranular fracture (secondary feature)
4, 5, 6	Annealed at 1280 °C	Vacuum	616.1	2.82	Cleavage fracture	Transgranular fracture (predominant) Intergranular fracture (slightly increased)
7, 8, 9	Annealed at 1280 °C	Air	579.5	2.65	Cleavage fracture	Transgranular fracture (predominant) Intergranular fracture (increase in proportion)
10, 11, 12	Annealed at 1280 °C	H_2_	531.1	2.49	Cleavage fracture	Mix of inter–and trans–lamellar fracture

**Table 5:** The performance degradation of Ti–45Al–8.5Nb–(0.2W, 0.2B, 0.02Y) alloys in hydrogen atmosphere compared with those in oxygen.

Material Condition	The Performance Degradation of Fracture Strength (%)	The Performance Degradation of ε_f_ (%)
As-cast	26.3	45.7
1240 °C	8.5	14.9
1280 °C	17.4	24.5

## Data Availability

The data used in this article are presented in the manuscript.
